# Cluster-Based Control Plane Messages Management in Software-Defined Flying Ad-Hoc Network

**DOI:** 10.3390/s20010067

**Published:** 2019-12-21

**Authors:** Pedro Cumino, Kaled Maciel, Thaís Tavares, Helder Oliveira, Denis Rosário, Eduardo Cerqueira

**Affiliations:** Computer Science Faculty, Federal University of Pará (UFPA), Belém 66075-110, Brazil; kaledmaciel@gmail.com (K.M.); thaistavares@ufpa.br (T.T.); heldermay@ufpa.br (H.O.); denis@ufpa.br (D.R.); cerqueira@ufpa.br (E.C.)

**Keywords:** control plane management, clustering, SDN-FANET, UAV contextual information

## Abstract

Collaboration between multiple Unmanned Aerial Vehicles (UAVs) to establish a Flying Ad-hoc Network (FANET) is a growing trend since future applications claim for more autonomous and rapidly deployable systems. In this context, Software-Defined Networking FANET (SDN-FANET ) separates the control and data plane and provides network programmability, which considers a centralized controller to perform all FANET control functions based on global UAV context information, such as UAV positions, movement trajectories, residual energy, and others. However, control message dissemination in an SDN-FANET with low overhead and high performance is not a trivial task due to FANET particular characteristics, i.e., high mobility, failures in UAV to UAV communication, and short communication range. With this in mind, it is essential to predict UAV information for control message dissemination as well as consider hierarchical network architecture, reducing bandwidth consumption and signaling overhead. In this article, we present a **C**luster-b**A**sed control **P**lane messages management in s**O**ftware-defined flying ad-hoc **NE**twork, called CAPONE. Based on UAV contextual information, the controller can predict UAV information without control message transmission. In addition, CAPONE divides the FANET into groups by computing the number of clusters using the Gap statistics method, which is input for a Fuzzy C-means method to determine the group leader and members. In this way, CAPONE reduces the bandwidth consumption and signaling overhead, while guaranteeing the control message delivering in FANET scenarios. Extensive simulations are used to show the gains of the CAPONE in terms of Packet Delivery Ratio, overhead, and energy compared to existing SDN-FANET architectures.

## 1. Introduction

Unmanned Aerial Vehicles (UAVs) are commonly used for autonomous missions, such as search and rescue missions [1], border surveillance [2], wildfire management [3], traffic monitoring [4], remote sensing [5], and other smart applications. Although single UAV systems have been used for decades, it is more advantageous to use a group of UAVs to establish Flying ad-hoc networks (FANETs) as it provides scalability, less energy consumption, lower loss, higher functionality [6,7]. In this sense, UAVs must be grouped in a properly coordinated manner to provide robust operation and to cover a larger area of interest [8]. FANETs can be used to capture different content, including video, from the event area, enabling humans (or even a service) in the control center to take appropriate action to explore a hazardous area, where rescuers are unable to reach easily and quickly [7,9]. This FANET will be the humans’ eyes in the sky, and UAVs must be flying and monitoring events in a coordinated fashion for a long period with reliability [10].

In FANET scenarios, UAVs must collaborate, and their behaviors (both the data transmission and the UAV movement) must be effectively controlled to maximize the FANET benefits and application performance [11]. In this way, a decentralized approach [6] to manage FANET include more complexity to synchronize information with all network nodes, requiring more control messages to be transmitted across the FANET, and can increase total power consumption. On the other hand, a centralized UAV controlling system can make optimized decisions based on the global UAV context information [12]. A centralized controller node must deal with the mobility trajectory of UAVs to avoid UAV collisions or improve application performance. It also needs to determine data routing paths, change packet transmission parameters (data rate or transmission power) due to performance or energy reasons. Hence, FANET could be managed by a Software Defined Network (SDN) [13] composed by a group of UAVs with a central controller entity [14]. In turn, SDN-FANET [12] implements the concept of SDN into FANET to separate the control and data plane, and to provide network programmability [15]. The FANET control plane programmability means to perform UAV placement and replacement, to improve the application performance, to determine optimal data routing paths, and other control tasks. The SDN-FANET controller considers the global UAV context information, such as UAV positions, movement trajectories, and residual energy, to perform all control functions. SDN-FANET provides flexibility to network management, where the network plane can be programmed to meet particular application requirements [16].

SDN-FANET controller requires accurate global UAV context information to prevent UAV collisions, optimize the UAVs’ movements, establish a routing path, and other decision making tasks [11]. In this way, the controller node must receive UAV context information with low packet loss to be aware of the UAV status for decision making, which is achieved by reliable control message dissemination [12]. However, control message dissemination with low overhead, and high performance is not a trivial task, due to particular characteristics of FANET, i.e., high mobility, failures in UAV to UAV communication, and short communication range [17]. The network topology changes quickly even if UAVs movements are controlled, leading to frequent control message dissemination, which increases the network overhead and interference, disconnection problems, increased latency, and packet losses [18]. In addition, FANETs are deployed for highly sensitive applications that require reliable transmission, but the SDN-FANET could share the same wireless medium for data and control messages. Under these circumstances, to keep FANET’s applications and services more reliable, stable, and active, it is essential to design an efficient and reliable control message transmission for FANET operations [19].

It is essential to predict UAV information without control message transmission, reducing bandwidth consumption, and signaling overhead. For instance, based on mobility or energy consumption prediction algorithms, the controller can change the frequency control message dissemination. Also, for large-scale environments, it might be required multi-hop communication to disseminate control messages, which can be achieved by deploying multiple local controllers that are responsible only for forwarding control messages to/from the central controller [11]. In this context, hierarchical network architecture, i.e., clustering, enhance the overall network performance of control messages management compared to flat architecture, by reducing energy consumption, interference, and packet loss, while increases scalability and application performance [20]. The hierarchical architecture provides better performance results in a broad mission area composed of a higher number of UAVs [6]. UAVs are organized into groups, and a set of UAVs are considered as Cluster-Head (CH), which work as a local controller to perform more complex tasks, such as the controlling of each group member, collecting data from non-CHs for data aggregation, and sending aggregated data to the controller [21]. It is essential to create groups based not only on placement but also on the residual energy of each node [6]. Besides, UAV mobility makes the network more dynamic to transmit data due to UAV mobility leads to communication flaws and void area. Thus, location, residual energy, and mobility prediction must be considered for an effective control message dissemination in FANET. However, to the best of our knowledge, there is not a unified solution that provides efficient control messages management together with a clustering algorithm.

In this article, we present a cluster-based control plane message management for SDN-FANET, called CAPONE (Cluster-bAsed control Plane messages management in sOftware-defined flying ad-hoc NEtwork). We introduce a new control message management approach for SDN based FANET by integrating acknowledgment, node location prediction, and a well known clustering approach. The controller is able to predict UAV information based on the UAVs contextual information without disseminating control messages. CAPONE divides the FANET into groups by considering two steps. Specifically, CAPONE determine the number of clusters using the Gap statistics method (i.e., step 1), which is input for a Fuzzy C-means method to determine the groups (i.e., step 2). For the control plane point of view, in each group, the CH works as a local controller to perform more complex tasks, while group members only send UAV contextual information based for the CH. In this sense, CAPONE minimizes the number of control messages without compromise application performance while increases the overall network lifetime. Simulation results show that CAPONE improves Packet Delivery Ratio (PDR), overhead, and remaining energy compared to state-of-the-art SDN-FANET architectures. The main contributions of this article are a cluster-based control message dissemination for SDN-FANET operations, efficient and reliable control message dissemination, implementation, and evaluation of CAPONE in a network simulator to assess network performance metrics in terms of PDR, position prediction error, overhead, and remaining energy.

We organize the rest of this article as follows. Section 2 outlines the state-of-the-art about control message dissemination for SDN-FANET, their main drawbacks for an efficient and reliable control message transmission for FANET operations. Section 3 describes the CAPONE protocol implemented in an SDN-FANET architecture. Section 4 details the simulation methodology and results used to evaluate CAPONE. Finally, Section 5 presents the concluding remarks and future work.

## 2. Related Work

Mazied et al. [22] highlighted the importance of control plane management in a wireless network for the 5G. In 5G, the SDN architecture is increasingly becoming employed to manage a wide range of heterogeneous wireless devices and software systems. In this context, the authors analyzed recent architectures suitable for 5G networks and have presented a qualitative comparison between the direct wireless connection and wireless indirect connection schemes. The authors designed an effective direct and indirect wireless SDN control plane framework to improve the management of heterogeneous devices in recent 5G networks.

Kumar et al. [23] proposed a secure mobility model between UAVs and wireless sensor network (WSN) nodes, where communication would be through the SDN controller for authentication and coordination. The authors exploit the advantages of SDN to support dynamic networks by establishing a central controller to manage the WSN coverage and authentication using UAVs. The UAVs trajectories are based on the density of each WSN cluster. This information is dynamically updated according to the statistics collected from the overall network.

Rametta and Schembra [24] present an SDN/NFV for FANET in a scenario where terrestrial nodes and UAVs are both equipped with cameras for monitoring rural areas. UAVs serve as a backbone network for the platform, where end-users monitor a particular rural area. The architecture comprises backbone drone nodes that form a meshed network, where UAVs are equipped with NFVs to provide virtual network functions (VNFs) that implement video transcoding and streaming. A central drone manages the network and controls all aspects of virtualization.

Kaleem and Rehmani [25] also have successfully applied an SDN architecture for FANET management. In this work, the authors present an amateur drone monitoring and tracking service as they might be a threat to citizens in a variety of scenarios. The proposed architecture is composed of a centralized controller present in clouds and UAV nodes responsible for monitoring, tracking, jamming, and hunting tasks.

Zhao et al. [12] implemented an SDN-FANET that uses SDN to separate the control and data plane to manage the UAVs in a coordinated manner/way. They focus on the problem of optimal UAV Relay Node placement for real-time video services and collision avoidance by considering global UAV context information, such as UAV position, movement trajectories, and residual energy. However, this work does not present a method to avoid network overhead once the number of control messages is not reduced.

Cumino et al. [11] introduced an SDN module to manage the UAV battery replenishment or replacement in scenarios of video surveillance. It takes advantage of an SDN architecture with a centralized controller to obtain the energy status of all UAVs and then decides when a node needs to go to a ground station to change or recharge its battery. This work considered that all the nodes have to send every second a beacon containing the location and energy status to the controller node. This way, the controller node is indeed always aware of the entire network, but the number of messages shall increase considerably with the increasing number of UAVs in different scenarios. In our work, one of the main goals is to reduce the amount of control messages transmissions in scenarios with clustered nodes.

To face FANET issues, such as communications issues due to frequently changing topology, Khan et al. [26] suggested a self-organized clustering scheme for FANET by using a behavioral study of Glowworm Swarm Optimization (GSO). In this case, the group formation and cluster head election consider the luciferin value and the residual energy of the UAVs and relies on the connectivity with a Ground Control Station (GCS). From a different perspective, we consider a centralized approach that takes advantage of a UAV as a Controller, which might attend larger and hard-to-reach areas.

Secinti et al. [15] proposed an SDN-FANET architecture that utilizes multiple wireless link access technologies. Furthermore, they devised a resilient multi-path routing protocol that identifies various disjoint routes for UAV pairs to improve the resiliency of the network. Moreover, their approach reduced the outage rate of end-to-end connections in the presence of malicious UAVs and achieved a moderate reduction in end-to-end delay compared to traditional algorithms. However, this work does not consider the battery limitations of the UAVs and the weight needed to carry different wireless link access technologies that could impact the performance of the UAVs.

Li et al. [27] implemented a dual-controller cooperative SDN-based FANET scheme and design a Dyna-Q-based reinforcement learning algorithm using power allocation and track planning collaborative optimization against smart jamming. The authors used two different controllers to keep track of the UAVs’ behavior and the other to be aware of the network status. In our proposal, we adopt the usage of a single controller responsible for managing the network as a whole, including the UAVs’ trajectories and their status.

Kirichek et al. [28] proposed an SDN-based solution for FANET to manage a group of UAVs for an Internet of Things (IoT) coverage. Flying nodes are used simply as switches to establish communication between terrestrial segments and a flying data collector. An additional node is considered as the SDN controller to update the other nodes’ routing tables. Their work showed the efficiency of the SDN-based solution through simulation.

Qi et al. [20] presented a centralized Traffic-Differentiated Routing (TDR). The protocol groups UAVs into several cluster domains. It runs on an SDN-based FANET and is executed in each cluster. Besides, it focuses on the data plane by guaranteeing the specific QoS requirements of delay-sensitive and performance-requisite applications. However, in our proposal, we provide reliable transmission of the management and coordination messages in the control plane, which leads to precise coordination and predictability of the UAVs positioning. Moreover, our work also highlights the method used to compute the clusters and choose the cluster heads.

Table 1 summarizes the main characteristics of existing works intended to provide control plane Message management for SDN-FANET. It compares the control plane message management for SDN-FANET described in this article to various others described in the literature concerning the application, routing approach, and control message transmission performance. Therefore, to the best of our knowledge, efficient control messages management together with a clustering algorithm has not yet been provided by a unified solution.

## 3. Cluster-Based Control Message Management for FANET

In this section, we introduce the Cluster-bAsed control Plane messages management in sOftware-defined flying ad-hoc NEtwork, called CAPONE. It aims to reduce the network overhead while guaranteeing the control message delivering in FANET scenarios. Based on UAV contextual information, the controller is able to predict UAV information without disseminating control messages. It divides the network into groups of UAVs to reduce the network overhead while guaranteeing the control message delivering in FANET scenarios. In this sense, CAPONE determine the number of clusters using the Gap statistics method, which is input for a Fuzzy C-means method to determine the groups. In this following, we present the main functionalities introduced by CAPONE, how the network operates during the clustering process, and how the nodes behave while inside a group.

### 3.1. Network and System Model

FANET can be used to monitor most kinds of environments without human intervention as it can reach hard areas, for instance, in disasters, emergency scenarios, buildings for structure inspection, and environmental surveillance [9]. In this sense, FANETs will be important in future smart city applications by enabling UAVs to collect measurements and to transmit for ground team or service, and thus UAVs will be the humans’ eyes in the sky [6,10]. It is because UAVs provide versatility, flexibility, and relatively small operating expenses to support autonomous actions, where rescuers are unable to reach easily and quickly. Data captured from the event area enable humans (or even a service) in the control center to take appropriate action based on rich information to explore a hazardous area, where rescuers are unable to reach easily and quickly [11]. For instance, multimedia data plays an essential role in helping ground rescue teams to make appropriate decisions based on detailed visual information [29].

We considered an SDN architecture applied to FANET, such as introduced by Zhao et al. [12]. In this context, SDN-FANET infrastructure is divided into three distinct planes (application, control, and forwarding planes), where each plane can be programmed to meet particular application requirements [12]. The application plane supports different FANET applications, such as surveillance, searching, wildlife tracking, video services, and others. The forwarding plane considers (re)configurable nodes (i.e., UAVs) connected to a centralized controller. The control plane has a controller node responsible to perform all control functions, such as collision manager, routing, topology management, UAVs replacement, and others. Specifically, the controller could be a ground station or a regular UAV node to collect UAV information and send configuration commands periodically [11,12].

We model the FANET as introduced by Cumino et al. [11]. The FANET is composed of *n* UAVs (nodes) with an unique identity (*i*∈[1,n]) monitoring an area of interest. We represent such UAVs considering a dynamic graph G(V,E). The vertices $V=\{v_{1},\cdots ,v_{n}\}$ represent a finite set of UAVs, and edges $E=\{e_{1},\cdots ,e_{n}\}$ means a finite set of asymmetric wireless links between 1-hop UAV $\left(v_{i}\right)$ neighbors [11]. The subset of all 1-hop UAV neighbors inside the radio range (RR) of a given UAV $v_{i}$ is denoted by $N(v_{i})\subset V$. We consider that each UAV $v_{i}$ has a queue (*Q*) in order to buffer packets at the routing and MAC layers, which has a maximum queue capacity ($Q_{max}$). Packets are removed from the queue to be sent considering First In First Out (FIFO) algorithm. On the other hand, packets are dropped using the Drop Tail algorithm in case of buffer overflow. We consider UAV $v_{i}$ equipped with a sensor unit to monitor the environment and a radio transceiver to send the collected data. For the convenience of notation, we denote Source Nodes ($SN_{i}\subset V$) as the subset of UAVs responsible for capturing data flows from the environment. The captured data is sent from the source nodes SN in a multi-hop fashion via multiple relay nodes ($RN_{i}$ ⊂ *V*), i.e., as the subset of UAVs responsible to forwarding data from the source node to the destination node. We assume a FANET scenario composed of one static DN∈V equipped with a radio transceiver and unlimited energy, which is responsible for receiving the collected for further processing and dissemination. A UAV $v_{i}$ can perform different tasks, so it can belong to different subset, however not at the same time. For instance, $v_{i}\in SN$ inffer that $v_{i}\notin RN$, and $v_{i}\notin DN$. Thus, RN≠SN≠DN during all the network life time. Each UAV $v_{i}$ is aware of its own location $L_{i,t}$ at a given timestamp *t* by means of a positioning system, e.g., GPS or Galileo. Each location $L_{i,t}$ is a 3-uple of geographical coordinates $\left(x_{i},y_{i},z_{i}\right)$ in a 3D space, since UAVs fly in a 3D space. On the other hand, the location of the DN is assumed to be known a priori by each node $v_{i}$, since it is a static node.

Each UAV $v_{i}$ flies with a given speed $s_{i}$ ranging between a minimum ($s_{min}$) and a maximum ($s_{max}$) speed limit towards a trajectory $traj_{i}$. The trajectory $traj_{i}$ is defined as an ordered sequence of locations that a given UAV $v_{i}$ flies between two pairs of location, which is denoted as $traj_{i}=$ {$L_{i,0},L_{i,1},\ldots ,L_{i,t}$ }, indicating that a given UAV $v_{i}$ arrives at location $L_{i,t}$ at timestamp *t*. Each UAVs $v_{i}$ flies according to the trajectories defined by the Paparazzi Mobility Model (PPRZM) [30], because PPRZM enables UAVs to adapt its trajectory for any mission based on different possible UAV movements [31]. Particularly, PPRZM is a stochastic mobility model based on autopilot software for UAV [32], which considers five possible trajectories ($traj_{i}$): Stay-At (i.e., UAV flies in a circle), Way-point (i.e., UAV flies following a straight line to a destination position), Eight (i.e., UAV trajectory has the 8 form around two fixed positions), Scan (i.e., UAV flies performs a scan in an area defined by two points along the round trip trajectories); and Oval (UAV trajectory has an oval form) [11]. Each movement defines a set of points for the UAV flies in straight trajectory $traj_{i}$ between two points (current and destination position) with a given speed $s_{i}$.

Each UAV $v_{i}$ has a battery with initial energy ($E_{v_{i}}\left(0\right)$), and it spends energy to transmit a packet ($E_{tx}$), to receive a packet ($E_{rx}$), to retrieve data ($e_{frame}$), and to fly with a given speed $s_{i}$ ($E_{fly}\left(d,s_{i}\right)$). Each UAV $v_{i}$ is able to measure the current energy ($E_{v_{i}}\left(t\right)$) at any time, and thus it can compute the remaining energy ratio ($E_{ratio_{v_{i}}}\left(t\right)$∈ [0, 1]) as follows:

(1) $$E_{ratio_{v_{i}}}\left(t\right)=\frac{E_{v_{i}}\left(t\right)}{E_{v_{i}}\left(0\right)}$$

$E_{fly}$ is the energy consumed in a straight flight to move a distance *d* at a given speed $s_{i}$. $E_{fly}$ can be computed as the integral of the power $P(s_{i})$ in function of $s_{i}$ along the time [33]:

(2) $$E_{fly}\left(d,s_{i}\right)=\int_{t=0}^{t=d/s_{i}}P\left(s_{i}\right)dt=P\left(s_{i}\right)\frac{d}{s_{i}}$$

The energy required to transmit ($E_{tx}$) and to receive ($E_{rx}$) a packet depends on the transmission power ($P_{tx}$). The energy consumed by the UAV $v_{i}$ to transmit a packet of length *n* bits over the wireless link with a bit rate equal to *r* bps in time *T* is computed as [34]:

(3) $$E_{tx}=\left(P_{ct}+\frac{P_{tx}}{e_{pa}}\right)T=\left(P_{ct}+\frac{P_{tx}}{e_{pa}}\right)\frac{n}{r}$$

where $P_{ct}$ is the power required to run the transmitting circuit, and $e_{pa}$ is the efficiency of the power amplifier. The energy consumed by the UAV $v_{i}$ to receive a packet $E_{rx}$ is computed based on Equation (4) [34]. The power required to run the receiving circuit is denoted as $P_{cr}$.

(4) $$E_{rx}=\frac{P_{cr}}{r}n$$

It should be highlighted that UAV movements require much more energy than for packet transmissions. In our scenario, we consider a battery replacement location $L(X_{r},Y_{r},Z_{r})$ that a given UAV $v_{i}$ could fly to replace/recharge the battery, and then return to the monitoring environment, as introduced by Cumino et al. [11].

**Figure 1:**
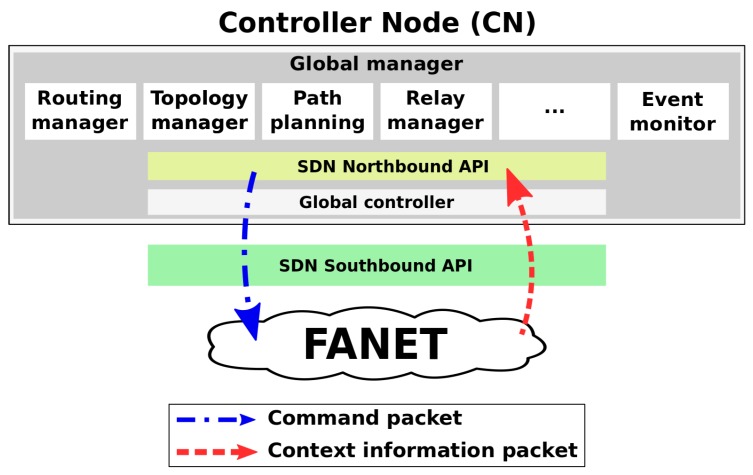
SDN-FANET control plane.

Figure 1 shows the control message flow in the control plane of the SDN-FANET architecture. The whole FANET is managed by a central controlling entity, known as Controller Node (CN⊂V). Controller (CN) is responsible for all management functions via the Global Manager and Global Controller. Specifically, controller CN must have full knowledge about each UAV $v_{i}$ contextual information, such as, remaining energy ratio $E_{ratio_{v_{i}}}\left(t\right)$, location information $L_{i,t}$, mobility trajectory $traj_{i}$, speed $s_{i}$, and others, for efficient decision-making [12]. Each UAV $u_{i}$ must send control message via SDN southbound API with contextual information $msg_{CI}$ to the Global Controller at controller CN. All the control messages are transmitted considering delivery guarantee to increase the reliability. Global Controller plays the role of an interface between the controller CN and FANET, synchronizing data exchange in both directions, requesting UAV contextual information, sending instructions to UAVs, and others [12].

Global Manager module performs all control functions, such as routing, topology management, path planning, relay manager, event monitor, and others, based on the inputs provided by the Global Controller module. For instance, the Global Manager module uses the UAV contextual information and the event location. It can establish a reliable routing path (by the routing manager module), place UAVs at the optimal locations to forward the packets (by the relay manager module), and how each UAV $v_{i}$ should move by considering the global topology information (by the path planning module). Then, the Global controller sends all the output decisions to the UAVs.

For large-scale environments, it might be required multi-hop communication to disseminate control message [6]. Some UAVs can be responsible only to forward the control message to/from the Controller CN, enabling to transmit control messages with lower transmission power, which leads to reduced interference and energy consumption, as well as better control message reliability [12]. In this context, hierarchical network architecture enhances the overall network performance of control messages management, where UAVs are organized into groups, and a set of UAVs are considered as CH to work as a local controller to perform more complex tasks, such as the controlling of each group members, collecting data from non-CHs for data aggregation, take local decisions, and sending aggregated data to the controller [21]. The controller node CN is responsible for creating the cluster, electing the CH, assign the cluster members, and disseminating this information. In the cluster operation, each cluster member sends contextual information to its CH, which performs data aggregation to avoid unnecessary data transmission. Afterward, each CH sends the aggregate data packets to the controller CN. Based on the received UAV context information, Controller CN considers mobility and energy prediction algorithms to reduce the contextual information dissemination. In the following section, we introduce the control message dissemination operations.

### 3.2. Control Message Dissemination Operations

Assuming the SDN architecture, the central controller CN is aware of every UAV $v_{i}$ contextual information, such as remaining energy ratio $E_{ratio_{v_{i}}}\left(t\right)$, current location $L_{i,t}$, mobility trajectory $traj_{i}$, speed $s_{i}$, and transmission and receiving ratio [11,12]. It supports an effective decision-making process to optimize overall FANET operations, for instance, how frequent a UAV disseminates its contextual information, which might change constantly depending on the current mobility and energy predictions calculated by CN. Besides, each UAV $v_{i}$ flies following a straight trajectory $traj_{i}$ between its current position $L_{i,t}$ and a destination position $L_{i,t+1}$ with a given speed $s_{i}$. To inform the CN about its conditions, $v_{i}$ sends control messages $msg_{CI}$ with its contextual information, including $L_{i,t}$, $L_{i,t+1}$, and $s_{i}$, to the controller CN just before leave $L_{i,t}$ and after arrive at $L_{i,t+1}$, reducing bandwidth consumption and signaling overhead. In this way, the communication improvement between UAV $v_{i}$ and controller CN considers an adaptive sending ratio.

The controller node CN receives the control messages $msg_{CI}$, and stores the contextual information in a corresponding table. The controller node CN is able to predict a given UAV location towards the trajectory $traj_{i}$ without the need of control message for location update, based on the current position $L_{i,t}$, destination position $L_{i,t+1}$, and flying speed of a given UAV $v_{i}$.

Similarly, CN is also able to estimate the required energy for $v_{i}$ based on the current transmission rate, receiving rate, and speed information, combined with the energy required for such tasks in, as shown in Equation (5). Equation (6) estimates the total energy that will be spent by a node based on the node’s current energy and the value calculated in Equation (5). In its turn, Equation (7) represents the remaining energy ratio estimation of $v_{i}$ for energy-aware decisions. For instance, UAV death can be detected by the controller CN based on the remaining energy ratio prediction.

(5) $$E_{spent}=E_{fly}\left(d,s_{i}\right)+E_{tx}+E_{rx}$$

(6) $$E_{v_{i}}\left(t+1\right)=E_{v_{i}}\left(t\right)-E_{spent}$$

(7) $$E_{ratio_{v_{i}}}\left(t+1\right)=\frac{E_{v_{i}}\left(t+1\right)}{E_{v_{i}}\left(0\right)}$$

However, the control messages $msg_{CI}$ must be transmitted with reliability and low delay to avoid inaccuracy prediction. For a more reliable transmission, the controller CN must send an ACK message after receiving the control message $msg_{CI}$ from a given UAV $v_{i}$. As soon as this ACK does not arrive at the UAV $v_{i}$ after time *t*, which is arbitrarily defined according to test evaluation, then the UAV $v_{i}$ sends the control message $msg_{CI}$ again. This process runs until the ACK message arrives at such UAV $v_{i}$. It is important to highlight that as soon as the controller CN does not receive a message $msg_{CI}$ from a given UAV $u_{i}$ informing that it reaches its destination location, the controller CN could search for such UAV $v_{i}$ location in the network by broadcasting a control message or wait for the message $msg_{CI}$ from the UAV $v_{i}$, where the former being the most ideal.

**Figure 2:**
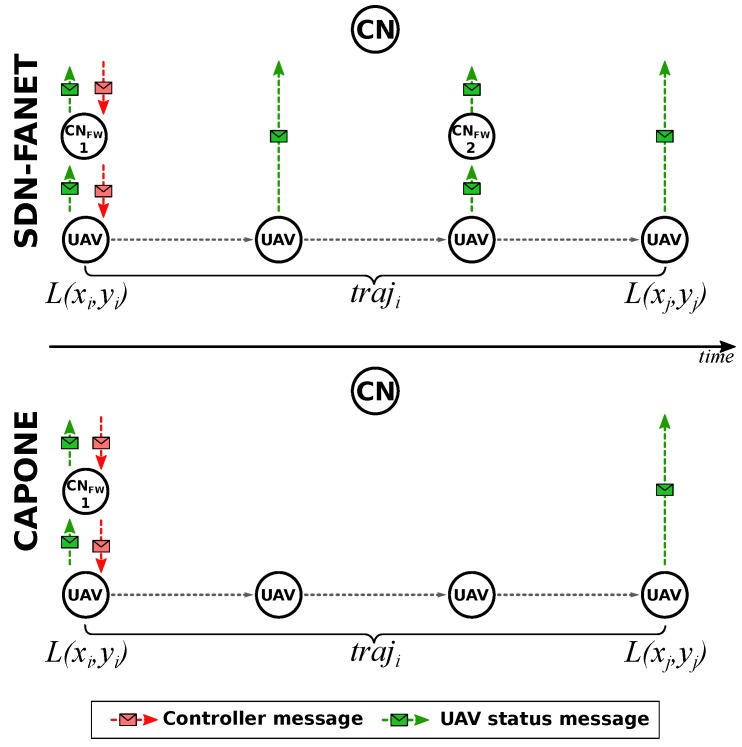
Control message transmission behavior of the network.

Figure 2 shows the UAV behavior for control message transmission considering SDN-FANET and CAPONE. As can be seen, a given UAV $v_{i}$ first sends its status to the controller, and, moves from location $L_{i,t}$ to location $L_{i,t+1}$ following the trajectory $traj_{i}$ after receives a beacon from the controller CN. The exchanged messages might be forwarded by a controller forwarder $CN_{fw}$, depending on how distant the UAV is from CN. The main differences between the two protocols are:

(i) In CAPONE, the UAV sends a single control message $msg_{CI}$ and then waits for the controller acknowledgment. The UAV sends more then one message only if it does not receive the acknowledgment after a certain amount of time. On the other hand, as SDN-FANET do not employ an acknowledgment packet, the UAV keeps sending information in every *t* time interval.
(ii) The UAVs follows the PPRZM mobility model. When a UAV is performing the Stay-at, Eight, or Oval movement pattern, it stays hovering in the same area, next to one or two close coordinates. In this case, CAPONE assumes that the UAV stays stationary in the same region. On the other hand, when the UAV is performing Way-point or Scan movement, CAPONE assumes that the UAV is moving between far distinct locations. In this case, the UAV sends its next location to the controller right before it moves, so that the controller can keep this information for future planning. However, in SDN-FANET, the UAVs send beacons without a previous decision-making process.
(iii) CAPONE employs a clustering approach to improve the control message management. The CN takes into consideration the location of each UAV and then selects the best candidates RNs to become CH. Each CH behaves temporarily as a controller forwarder ($CN_{fw}$), connecting the CN to the other UAVs. The CHs changes according to position configuration of all UAVs.

### 3.3. Cluster-Based Operations

For large-scale environments, it might be required multi-hop communication to disseminate control message. Thus, we consider a hierarchical network architecture, where UAVs are organized into groups with a set CH (i.e., local controller) to perform more complex tasks, such as, controlling of each group members, collecting data from non-CHs for data aggregation, forwarding control messages to/from the controller node CN, take local decision, and sending aggregated data to the controller. In this way, we introduce a clustering-based algorithm to define a suitable position for a set of $CN_{fw}$, which behaves as CH. Moreover, the number of forwarders $n_{fw}$ is adjusted by considering UAVs’ location. The approach makes use of a partition-based clustering algorithm to allocate $n_{fw}$ forwarder nodes $CN_{fw}$ every time interval $T_{fw}$.

The implemented clustering method considers two well-known algorithms: Fuzzy C-means [35] for clustering and the Gap statistics [36] to define the number of clusters. Specifically, Gap statistics estimates an optimal number of clusters by finding a way to standardize the comparison with a null reference distribution of a data, i.e., a distribution with no obvious clustering. On the other hand, Fuzzy C-means allows one piece of data to belong to two or more clusters. To this extent, a UAV might be attended by more than one forwarder node, and it is up to the UAV do decide its next-hop address by simply evaluating the signal strength of each $CN_{fw}$. The different membership degree provided by Fuzzy C-means for each group enables to change the groups easily and quickly, in case a CH could fail, or a UAV could move closer to other CH.

The first step is to iterate over predefined cluster numbers ($c\in \left[1,2,3,\ldots ,C\right]$) computing the Fuzzy C-means objective function $J_{c}$ based on Equation (8), which gives a measure of the compactness of the first clustering. It considers the cluster index *c*, the membership coefficient $\mu_{ic}^{m}$, the *fuzzification* index *m* (m∈R, with m>1) to control the shape of membership functions, and the Euclidean distance $D_{ic}^{2}$ between the $i^{th}$ object and the $c^{th}$ cluster center. In addition, we consider a predefined set of random positions *B*, which the objective function $J_{c,b}^{*}$ is calculated for each set ($b\in \left[1,2,3,\ldots ,B\right]$).

(8) $$J=\sum_{i=1}^{N}\sum_{c=1}^{C}\mu_{ic}^{m}D_{ic}^{2}$$

The Gap statistics (Gap(c)) function compares the objective functions of the sets B and the original positions, which is computed based on Equation (9). It returns the information of how organized the positions are for each cluster number *c*, compared to a set of disorganized positions. In light of this, the clustered index maximizes the value of this function, should give a good approximation of the cluster number to be used.

(9) $$Gap\left(c\right)=\left(\frac{1}{B}\right)\sum_{b}\log\left(J_{c,b}^{*}\right)-\log\left(J_{c}\right)$$

Gap statistics (Gap(c)) function usually returns an average gap value, since it is not an exact method. For an accurate view of the value returned from Equation (9), the standard deviation (sd(c)) for each cluster number *c* is computed based on Equation (10).

(10) $$sd\left(c\right)=\sqrt{\frac{\sum_{b}{\left(\log\left(J_{c,b}\right)-\frac{1}{B}\sum_{b}\log\left(J_{c,b}^{*}\right)\right)}^{2}}{B}}$$

The intersection between the simulation error s(c), represented in Equation (11), and the maximum values of Gap(c) is computed, and the smallest value from *c* is defined as the optimal number of clusters estimated.

(11) $$s\left(c\right)=sd_{c}\sqrt{1+B^{-1}}$$

A fuzzy clustering is a collection of *k* clusters, $c_{1},c_{2},\ldots ,c_{k}$, and a partition matrix $M=m_{i,j}\in \left[0,1\right]$, for i=1…n and j=1…k, where each element $m_{i,j}$ is a membership indicator that represents in what degree an object *i* belongs to a cluster $c_{j}$. Fuzzy C-means algorithm runs towards the minimization of the objective function Equation (8). Its input is the number of clusters returned by the Gap statistics. Then, assigns coefficients randomly to each point for being in the clusters. And finally, it computes the centroid of each cluster and the new membership index of each point until the centroid stops change. Algorithm 1 describes the whole process, which is computed at each time interval at the controller node CN. In this way, the controller node CN is responsible for creating the cluster, electing the CH, assign the cluster members, and disseminating the defined clusters and CH.

Each cluster member sends contextual information to its CH, which performs data aggregation to avoid unnecessary data transmission. Afterward, each CH sends the aggregate data packets to the controller CN. In this way, each CH works as a local controller to perform more complex tasks, such as the controlling of each group members, collecting data from non-CHs for data aggregation, take the local decision, sending aggregated data to the controller, forward control messages to/from the controller node CN.
**Algorithm 1:** Fuzzy C-means
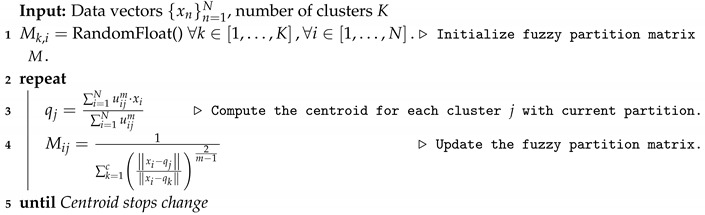


## 4. Evaluation

In this section, we present the simulations to demonstrate the efficiency of the CAPONE in transmitting control messages over FANET. Specifically, we present the methodology and metrics applied to evaluate the CAPONE for the management of control messages to support the operations of surveillance applications in a disaster recovery scenario. We evaluated the impact of different existing protocols on the PDR, position prediction error, overhead, and remaining energy.

### 4.1. Simulation Description and Metrics

We consider the Mobile Multi-Media Wireless Sensor Network (M3WSN) OMNeT++ framework [37] for our evaluation, which implements network stack for FANET communications as introduced in Section 3.1. We conducted 33 simulation runs with different randomly generated seeds, as it statistically provides a reliable behavior analysis of the proposal [38], and results show the values with a confidence interval of 95% [11,12]. We set the simulation time as 300 seconds (s), and simulation parameters to allow wireless channel temporal variations, link asymmetry, and irregular radio ranges, as expected in a real FANET scenario [11]. The FANET is composed of 34 UAVs flying with speed raging between 5 and 10 m/s over the entire flat terrain of 200 × 200 m [17] to explore and disseminate live video streaming from the environment. The FANET has one fixed Destination Node (DN) located at (100,0), as expected in FANET multimedia applications, such as safety & security, environmental monitoring, and natural disaster recovery [9]. We consider one UAV located at (100,100), working as controller node CN to periodically collect UAV information and send configuration commands [11].

In terms of FANET operations, we scheduled a random event at a different location, where UAVs are monitoring and sending information from the environment. Based on the collected information, the controller node CN could detect an event (based on the event monitor module in the Global Manager) at a given location, and then establishes a route between source SN and destination DN nodes via multiple relay nodes $RN_{i}$ based on UAV contextual information and the event location (based on the routing manager module in the Global Manager) [11]. For video application, we considered video sequences with different video features downloaded from the YUV video trace library and YouTube [39], where such videos are encoded using H.264 codec at 300 kbps, 30 fps, Group of Pictures (GoP) size of 20 frames, and common intermediate format (352 × 288 pixels) [11].

In the simulation, UAVs have a battery with an initial energy of 18’720 Joules [11] and are equipped with IEEE 802.11g with a nominal radio range $R_{max}$ of 55 m [18,40]. At the MAC layer, we consider the CSMA/CA MAC protocol without using RTS/CTS messages and retransmissions. At the application layer, we consider the QoE-aware redundancy mechanism to add redundant packets only to priority frames [17]. We have conducted simulations with three different control plane message management mechanisms, namely SDN-FANET, SDN-FANET + PREDICTION, and CAPONE. Particularly, SDN-FANET considers the SDN paradigm applied to FANET, which considers a flat network architecture with periodic transmissions of global UAV contextual information. On the other hand, SDN-FANET + PREDICTION also considers a flat network architecture with the control message dissemination operations introduced in Section 4.2. Finally, CAPONE improve the control messages transmission performance by predicting the positions of the UAVs, and considers hierarchical network architecture, as introduced in Section 3. For all proposals, we are evaluating the PDR, position prediction error, overhead, and remaining energy.

- PDR (%): The ratio between the number of control messages sent by UAVs and the number of delivered control messages at the controller node CN.
- Position prediction error (meters): Error between the UAV position at the controller node CN and the current UAV position at each time interval.
- Overhead: Total number of control messages $msg_{CI}$ transmitted with UAV contextual information during the entire simulation.
- Remaining energy (%): The ratio between the current energy level and the initial energy level by each UAV.

### 4.2. Simulation Results

**Figure 3:**
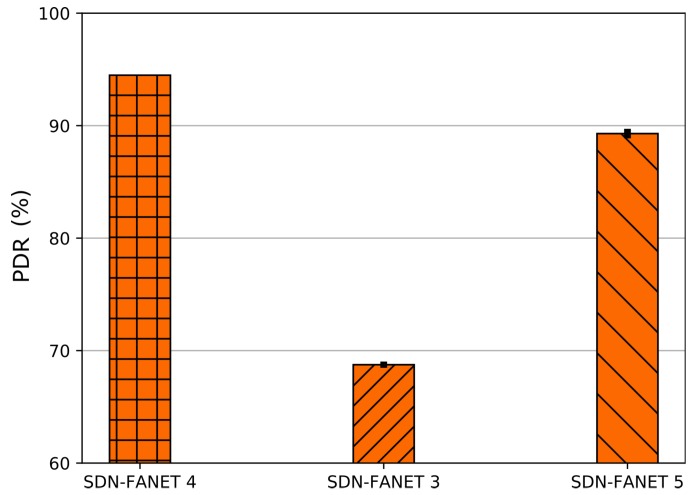
PDR considering different number of forwarder nodes for SDN-FANET.

SDN-FANET [12] considers multi-hop communication to disseminate control messages by deploying a constant number of forwarder node $CN_{fw}$. These forwarder nodes are $CN_{fw}$ hovering on fixed positions to guarantee the connection range between the CN and distant UAVs. To better define the number of forwarding nodes ($CN_{fw}$) in SDN-FANET, we analyze the PDR by varying the number of forwarding nodes $CN_{fw}$ distributed around the controller nod in the scenario, i.e., 3, 4, and 5. By analyzing the results, we achieved better PDR using 4 forwarding nodes $CN_{fw}$. The results depicted in Figure 3 shows PDR using different number of static controller forwarder $CN_{fw}$. Based on the results of Figure 3, it is possible to conclude that 4 controller forwarder $CN_{fw}$ provided the highest PDR for control message dissemination in SDN-FANET operations. This is because the forwarder nodes are equally distributed around the CN in static positions forming a square with a fixed distance. Besides, the distance between a $CN_{fw}$ and the edge of the scenario corresponds to the maximum transmission range that a UAV can reach. Considering 3 forwarder node $CN_{fw}$, some UAVs are not able to reach the controller node CN, i.e., caused by void area. In addition, 5 or more forwarder node $CN_{fw}$ increases the number of hops and the interference caused by forwarding tasks, which causes more collisions at the MAC layer and increases the delay, where control messages with high delay are dropped.

**Figure 4:**
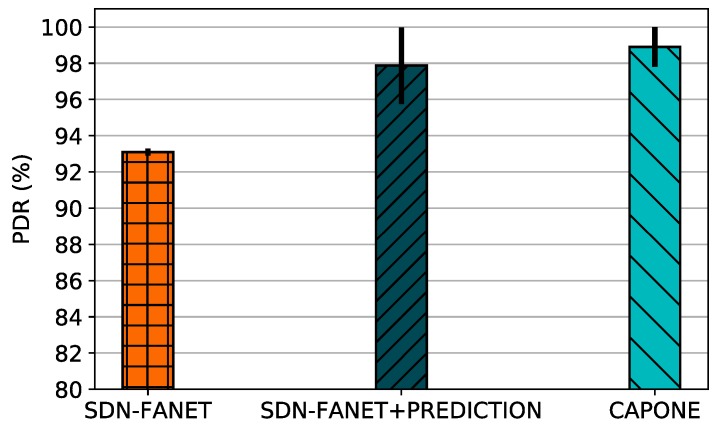
PDR for different control plane message management mechanisms.

Figure 4 shows the PDR for control message dissemination considering different mechanisms. As shown in Figure 4, CAPONE and SDN-FANET + PREDICTION deliver more packets compared to SDN-FANET in most cases. Both reduce the number of transmitted control messages by considering a prediction algorithm. However, it is important to highlight that the standard deviation of CAPONE is lower than SDN-FANET + PREDICTION, since CAPONE considers a clustering approach, where the CH is responsible for control packets aggregation to reduce the network overhead. On the other hand, SDN-FANET considers a continuous and periodic control message transmission, causing more collisions at the MAC layer and increasing the delay, where the delayed packets are dropped, such as explained for the results of Figure 3.

**Figure 5:**
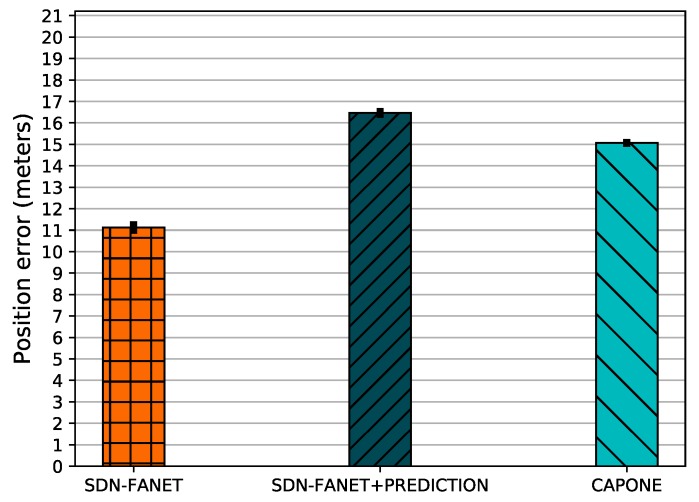
Position error for different control plane message management mechanisms.

Figure 5 shows the position error considering different control plane message management mechanisms. By analyzing the results, we conclude that SDN-FANET has a lower position error compared to SDN-FANET + PREDICTION and CAPONE. This is because in SDN-FANET, as the messages are continuously sent by all the nodes every second, it is more likely that the controller node CN receives a more accurate location. On the other hand, SDN-FANET + PREDICTION and CAPONE considers mobility prediction to reduce the contextual information dissemination, which introduces an acceptable error for decision making in FANET scenarios.

**Figure 6:**
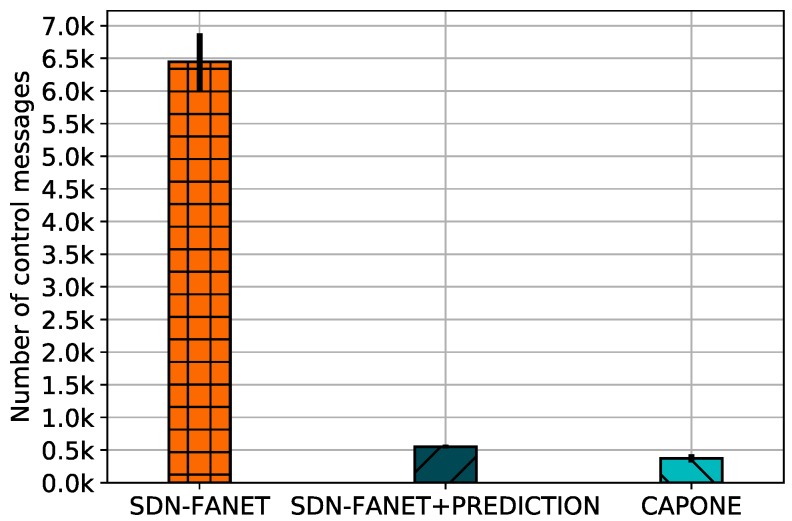
Overhead for different control plane message management mechanisms.

Figure 6 shows the total number of control messages transmitted by all UAVs during the entire simulation i.e., overhead, introduced by different control plane message management mechanisms. By analyzing the results, we can conclude that UAVs considering SDN-FANET transmitted about 6500 control messages during its operation, due to its constant control message transmissions. The prediction approach considered by SDN-FANET + PREDICTION decreased this value to approximately 550 messages by only send data when the UAV considerably changes its position, while CAPONE obtained the best result, transmitting about 390 messages during its operation. CAPONE reduces the network overhead due to its predicting and clustering approach, making the communication process more efficient.

**Figure 7:**
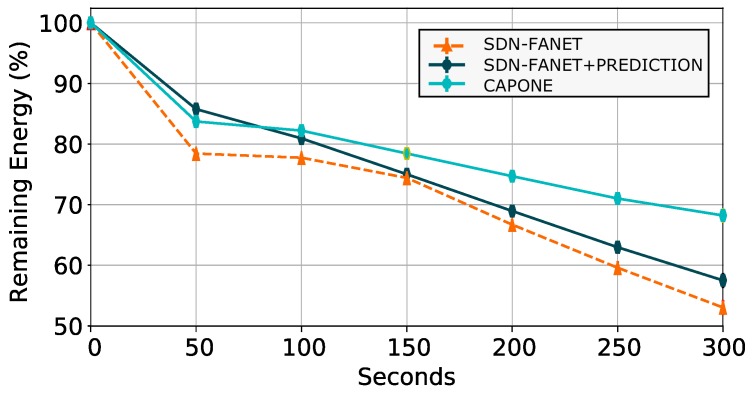
Remaining energy during the simulation for different control plane message management mechanisms.

Figure 7 shows the average remaining energy in each node considering evaluated SDN-FANET architectures. By analyzing the results, we conclude that CAPONE saved 25% and 14% of energy compared to SDN-FANET and SDN-FANET + PREDICTION, respectively, at the end of 300 simulation seconds. This is because SDN-FANET considers periodic control message dissemination, which consumes more energy for nodes that are sending and receiving the message. On the other hand, SDN-FANET + PREDICTION considers only prediction algorithms to reduce the number of control message transmissions. Finally, CAPONE considers a clustering approach and prediction algorithms to reduces the required number of control messages for SDN-FANET operations.

## 5. Conclusions and Future Work

This article presented CAPONE, a cluster-based control plane management protocol that reduces the network overhead while guaranteeing the UAV management and control message delivering in an SDN-FANET. The controller node considers the UAV contextual information to predicts the UAV movements without the need for constant control message transmissions, reducing bandwidth consumption and signaling overhead. The prediction and acknowledgment mechanisms not only reduce the number of control packets in the network but also improves the control message transmission as less packet occupies the network bandwidth.

CAPONE also divides the network into groups of UAVs while guaranteeing the control message delivering in FANET scenarios. It shows considerable advantages in terms of PDR, energy consumption, and overhead, even with a slight reduction of position accuracy. CAPONE determines the number of clusters using the Gap statistics method. This technique defines the best number of clusters according to the current locations of the UAVs. Fuzzy C-means method uses the number of clusters as input, defines the cluster heads, and groups the nodes of each cluster. It allows the controller to consider an association or membership degree for each node, ensuring communication between a UAV and the controller node.

For future work, we are planning to consider more dynamic and heterogeneous scenarios, with different kind of data source nodes, such as vehicles, ground users, and others. Moreover, the drawback of a single point of failure, usually present in a centralized approach, shall be attenuated with more controllers in the network. Thus, we are planning to define the best number of controllers for different scenarios and analyze the impacts on the network in terms of control management and service provisioning.

## Figures and Tables

**Table 1:** Summary of SDN-FANET proposals and integrated features.

Proposal	Application	Routing Approach	Control Performance
Mazied et al. [22]	General 5G applications	Wireless multi-path routing	✕
Kumar et al. [23]	Coverage and trajectory planning	Not specified	✕
Rametta and Schembra [24]	Video dissemination	Not specified	✕
Kaleem and Rehmani [25]	Monitoring and tracking	Not specified	✕
Zhao et al. [12]	Video dissemination	Not specified	✕
Cumino et al. [11]	Video dissemination	Not specified	✕
Khan et al. [26]	Varied Bit Rate traffic	Clustering supported by GSO	✓
Secinti et al. [15]	Jamming attack	Dijkstra with Vertex splitting	✕
Li et al. [27]	Jamming attack	Not specified	✕
Kirichek et al. [28]	IoT coverage and data gathering	Reactive with broadcast packets	✓
Qi et al. [20]	Varied Bit Rate traffic	Based on Ant Colony	✓
CAPONE	Video dissemination	Fuzzy C-means and GAP	✓

## References

[1] Dinh T.D., Pirmagomedov R., Pham V.D., Ahmed A.A., Kirichek R., Glushakov R., Vladyko A. (2019). Unmanned aerial system—Assisted wilderness search and rescue mission. Int. J. Distrib. Sens. Netw..

[2] Al Fayez F., Hammoudeh M., Adebisi B., Abdul Sattar K.N. (2019). Assessing the effectiveness of flying ad hoc networks for international border surveillance. Int. J. Distrib. Sens. Netw..

[3] Lin Z., Liu H.H., Wotton M. (2018). Kalman filter-based large-scale wildfire monitoring with a system of UAVs. IEEE Trans. Ind. Electron..

[4] Liu X., Peng Z.R., Zhang L.Y. (2019). Real-time UAV Rerouting for Traffic Monitoring with Decomposition Based Multi-objective Optimization. J. Intell. Robot. Syst..

[5] Zhu X., Meng L., Zhang Y., Weng Q., Morris J. (2019). Tidal and Meteorological Influences on the Growth of Invasive Spartina alterniflora: Evidence from UAV Remote Sensing. Remote Sens..

[6] Bekmezci I., Sahingoz O.K., Temel Ş. (2013). Flying Ad-hoc Networks (FANETs): A Survey. Ad Hoc Netw..

[7] Yanmaz E., Yahyanejad S., Rinner B., Hellwagner H., Bettstetter C. (2018). Drone networks: Communications, coordination, and sensing. Ad Hoc Netw..

[8] Gupta L., Jain R., Vaszkun G. (2016). Survey of Important Issues in UAV Communication Networks. IEEE Commun. Surv. Tutor..

[9] Arnaldo Filho J., Rosário D., Rosário D., Santos A., Gerla M. (2018). Satisfactory Video Dissemination on FANETs based on an Enhanced UAV Relay Placement Service. Ann. Telecommun..

[10] Erdelj M., Saif O., Natalizio E., Fantoni I. (2017). UAVs that Fly Forever: Uninterrupted Structural Inspection Through Automatic UAV Replacement. Ad Hoc Netw..

[11] Cumino P., Lobato Junior W., Tavares T., Santos H., Rosário D., Cerqueira E., Villas L., Gerla M. (2018). Cooperative UAV Scheme for Enhancing Video Transmission and Global Network Energy Efficiency. Sensors.

[12] Zhao Z., Cumino P., Souza A., Rosário D., Braun T., Cerqueira E., Gerla M. (2018). Software-Defined Unmanned Aerial Vehicles Networking for Video Dissemination Services. Ad hoc Netw..

[13] Wickboldt J.A., De Jesus W.P., Isolani P.H., Both C.B., Rochol J., Granville L.Z. (2015). Software-Defined Networking: Management Requirements and Challenges. IEEE Commun. Mag..

[14] Zhao Z., Schiller E., Kalogeiton E., Braun T., Stiller B., Garip M.T., Joy J., Gerla M., Akhtar N., Matta I. (2017). Autonomic Communications in Software-Driven Networks. IEEE J. Sel. Areas Commun..

[15] Secinti G., Darian P.B., Canberk B., Chowdhury K.R. (2018). SDNs in the Sky: Robust End-to-End Connectivity for Aerial Vehicular Networks. IEEE Commun. Mag..

[16] Zacarias I., Gaspary L.P., Kohl A., Fernandes R.Q., Stocchero J.M., de Freitas E.P. (2017). Combining Software-Defined and Delay-Tolerant Approaches in Last-Mile Tactical Edge Networking. IEEE Commun. Mag..

[17] Rosário D., Zhao Z., Santos A., Braun T., Cerqueira E. (2014). A Beaconless Opportunistic Routing based on a Cross-layer Approach for Efficient Video Dissemination in Mobile Multimedia IoT Applications. Comput. Commun..

[18] Guillen-Perez A., Cano M.D. (2018). Flying Ad Hoc Networks: A New Domain for Network Communications. Sensors.

[19] Khan M., Khan I., Safi A., Quershi I. (2018). Dynamic routing in flying ad-hoc networks using topology-based routing protocols. Drones.

[20] Qi W., Song Q., Kong X., Guo L. (2017). A traffic-differentiated routing algorithm in Flying Ad Hoc Sensor Networks with SDN cluster controllers. J. Franklin Inst..

[21] Thammawichai M., Baliyarasimhuni S.P., Kerrigan E.C., Sousa J.B. (2017). Optimizing communication and computation for multi-UAV information gathering applications. IEEE Trans. Aerosp. Electron. Syst..

[22] Mazied E.A., ElNainay M.Y., Abdel-Rahman M.J., Midkiff S.F., Rizk M.R., Rakha H.A., MacKenzie A.B. (2019). The wireless control plane: An overview and directions for future research. J. Netw. Comput. Appl..

[23] Kumar R., Sayeed M.A., Sharma V., You I. (2017). An SDN-Based Secure Mobility Model for UAV-Ground Communications. Mobile Internet Security. MobiSec 2017. Communications in Computer and Information Science.

[24] Rametta C., Schembra G. (2017). Designing a softwarized network deployed on a fleet of drones for rural zone monitoring. Future Internet.

[25] Kaleem Z., Rehmani M.H. (2018). Amateur drone monitoring: State-of-the-art architectures, key enabling technologies, and future research directions. IEEE Wirel. Commun..

[26] Khan A., Aftab F., Zhang Z. (2019). Self-organization based clustering scheme for FANETs using Glowworm Swarm Optimization. Phys. Commun..

[27] Li Z., Lu Y., Shi Y., Wang Z., Qiao W., Liu Y. (2019). A Dyna-Q-Based Solution for UAV Networks Against Smart Jamming Attacks. Symmetry.

[28] Kirichek R., Vladyko A., Paramonov A., Koucheryavy A. Software-defined architecture for flying ubiquitous sensor networking. Proceedings of the 2017 19th International Conference on Advanced Communication Technology (ICACT).

[29] Pimentel L., Rosário D., Seruffo M., Zhao Z., Braun T. (2015). Adaptive Beaconless Opportunistic Routing for Multimedia Distribution. Wired/Wireless Internet Communication.

[30] Bouachir O., Abrassart A., Garcia F., Larrieu N. A Mobility Model for UAV Ad Hoc Network. Proceedings of the International Conference on Unmanned Aircraft Systems (ICUAS).

[31] Hayat S., Yanmaz E., Muzaffar R. (2016). Survey on Unmanned Aerial Vehicle Networks for Civil Applications: A Communications Viewpoint. IEEE Commun. Surv. Tutor..

[32] Brisset P., Drouin A., Gorraz M., Huard P.S., Tyler J. The paparazzi solution. Proceedings of the 2nd US-European Competition and Workshop on Micro Air Vehicles.

[33] Di Franco C., Buttazzo G. Energy-aware Coverage Path Planning of UAVs. Proceedings of the IEEE International Conference on Autonomous Robot Systems and Competitions (ICARSC).

[34] Vazifehdan J., Prasad R.V., Jacobsson M., Niemegeers I. (2012). An Analytical Energy Consumption Model for Packet Transfer over Wireless Links. IEEE Commun. Lett..

[35] Bezdek J.C. (2013). Pattern Recognition with Fuzzy Objective Function Algorithms.

[36] Tibshirani R., Walther G., Hastie T. (2001). Estimating the number of clusters in a data set via the gap statistic. J. R. Stat. Soc. Ser. B Stat. Methodol..

[37] Rosário D., Zhao Z., Silva C., Cerqueira E., Braun T. An OMNeT++ Framework to Evaluate Video Transmission in Mobile Wireless Multimedia Sensor Networks. Proceedings of the International Workshop on OMNeT++ (ICST).

[38] Bukh P.N.D. (1991). The Art of Computer Systems Performance Analysis, Techniques for Experimental Design, Measurement, Simulation and Modeling.

[39] GERCOM Videos Used for Simulations. http://www.gercom.ufpa.br/index.php?option=com_jdownloads&view=category&catid=8&Itemid=343&lang=br.

[40] Shinkuma R., Mandayam N.B. (2018). Design of Ad Hoc Wireless Mesh Networks Formed by Unmanned Aerial Vehicles with Advanced Mechanical Automation. arXiv.

